# Protocol for quantification of fungal biomass in fungal composite materials based on ergosterol extraction

**DOI:** 10.1016/j.xpro.2026.104858

**Published:** 2026-09-24

**Authors:** Carsten Freidank-Pohl, Antonia Geyer, Ulla Simon

**Affiliations:** 1 Chair of Applied and Molecular Microbiology, Institute of Biotechnology, Technische Universität Berlin, Straße des 17. Juni 135, 10263 Berlin, Germany; 2 Chair of Advanced Ceramic Materials, Institute of Material Science and Technology, Technische Universität Berlin, Straße des 17. Juni 135, 10623 Berlin, Germany

**Keywords:** Metabolism, Microbiology, Biotechnology and bioengineering

## Abstract

Fungal composite materials are lignocellulosic particles and possible inorganic additives, connected by fungal hyphae. Here, we provide a protocol for extracting the cell membrane constituent ergosterol from dried fungal composite material to quantify the fungal fraction within the fungal composite material. We describe steps for preparing calibration samples and solvent-based ergosterol extraction. This protocol can be applied to quantify fungal abundance inside fungal composite material, providing the amount of fungal biomass as a novel parameter for a more in-depth material characterization.

## Highlights

• Considerations for target molecule selection to quantify fungal abundance
• Instructions for preparing fungal reference biomass samples
• Procedures for extracting ergosterol from fungal composite materials

## Abstract (editor highlights)

Publisher’s note: Undertaking any experimental protocol requires adherence to local institutional guidelines for laboratory safety and ethics.

## Graphical abstract

**Figure:**
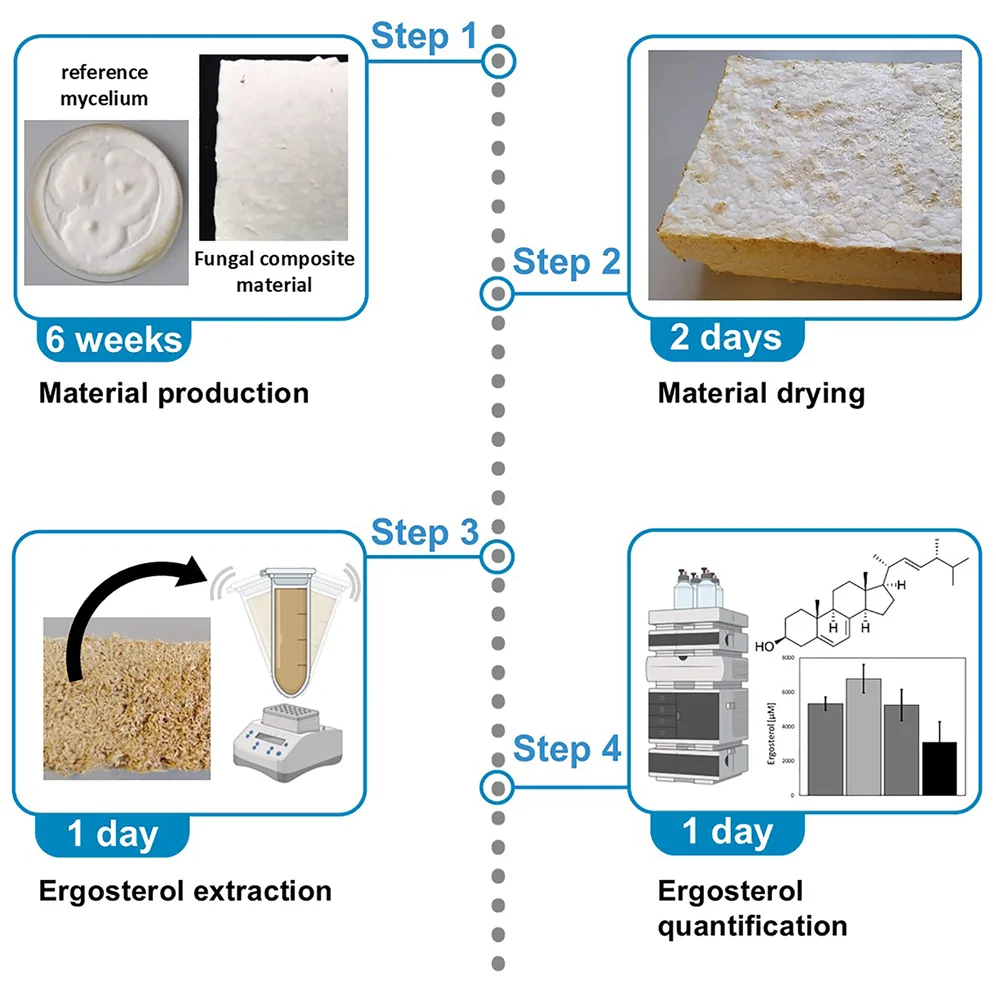

## Teaser

Fungal composite materials are lignocellulosic particles and inorganic additives, connected by fungal hyphae. Here, we provide a protocol for extracting the cell membrane constituent ergosterol from dried fungal composite material to quantify the fungal fraction within the fungal composite material. We describe steps for preparing calibration samples and solvent-based ergosterol extraction. This protocol can be applied to quantify fungal abundance inside fungal composite material, providing the amount of fungal biomass as a novel parameter for a more in-depth material characterization.

## Before you begin

Fungal composite materials and engineered living materials from fungi are emerging fields of research that utilize the growth of fungal organisms on organic and/or inorganic particles to generate materials. In this context, measuring the abundance of fungal biomass is often perceived as challenging but is an important parameter required for full characterization of the produced materials, as fungal mycelium growth is commonly not homogenously distributed from the surface to the composites’ inner part. This is usually caused by gradients of oxygen availability inside the material. Since optical quantification cannot be applied due to the high particle burden, biochemical assays detecting unique fungal biomarkers are required.

The major components of the fungal cell wall are chitin, forming the lower part of the cell wall adjacent to the cell membrane, β-1,6-glucan and, to a lesser extent, α-1,3-glucan. The fungal cell wall is highly specific for each fungal species.^1^ While fungal β-glucans can be determined with enzymatic assays^2^ combining acid hydrolysis with sulfuric acid and enzymatic conversion to glucose using a mixture of exo-1,3-β- glucanase and β-glucosidase, using glucans as a marker of fungal abundance, is impossible due to the frequent occurrence of plant glucans in fungal composite materials. The quantification of chitin also relies on the hydrolysis of the polymer into N-Acetylglucosamine (GlcNAc) which is often realized by acid treatments of the fungal biomass whereby deacetylation occurs that produces Glucosamine. Whilst GlcNAc is only quantifiable by GC-MS,^3^ Glucosamine can also be quantified colorimetrically^4^ in microtiter plates. However, a limitation of using chitin as a proxy for fungal biomass abundance is the presence of GlcNAc in the bacterial cell wall, hampering exact quantification in mixed cultures. Both chitin and the fungal glucan content in the cell wall can vary considerably during growth,^5^ adaption to different stressors^6^ and environmental conditions,^7^ increasing the thickness of the fungal cell wall by several 10 nanometers. Thus, quantification of both biopolymers is considered to become biased by the growth stage of the fungus, the environment and the age of the culture which can differ inside the fungal composite material. Additionally, the protocols for acid hydrolysis of the fungal cell wall are time consuming.

As an alternative, the quantification of fungal DNA in biomaterial samples has been recently proposed as a strategy to quantify fungal growth inside composite samples.^8^ The isolation success of genomic DNA from complex samples can vary significantly and might require extensive optimization that can be strain dependent.

In contrast, ergosterol is a unique constituent of the fungal cell membrane and thereby making it an interesting target to quantify the abundance of fungal biomass in fungal composite materials and engineered living materials. Ergosterol is successfully established as a marker for the abundance of fungi in ecology,^9^^,^^10^ quantifying fungal growth in solid-state fermentations of foods^11^ and fungal composite materials.^12^^,^^13^ Whilst older extraction protocols for ergosterol relied on the combination of NaOH and MeOH for saponification of lipids which requires extensive sample cleanup by solid-phase-extraction before HPLC analysis,^10^ we here use a combination of chloroform and methanol which has proven more efficient for ergosterol extraction from plant.^14^^,^^15^ Although ergosterol represents a unique marker for fungal organisms, ergosterol content in the cell membrane can also vary between cultivation conditions. Sterol homeostasis in fungi is regulated by the transcription factor Upc2^16^ which is translocated to the nucleus via Hsp90 when not binding to intercellular ergosterol.^17^

We here provide a step-by-step protocol for extraction of ergosterol to users new to the field of fungal composite materials, including the instrumentational requirements as well as the considerations of sample volumes and controls to be included. This protocol is relatively fast to accomplish, allows for processing a high number of samples in parallel and enables the quantification of fungal biomass inside fungal composite materials.

### Innovation

Our protocol introduces the use of a reference cultivation on solid agar supplemented with the same ratio of lignocellulosic materials used in the final composite material production process. Thereby, an over- or underestimation of ergosterol levels in fungal composite samples is minimized. Reduction of this cultivation bias is critical for accurate quantification of fungal biomass and whether this impacts the material performance. Additionally, we also raise attention to the user that substrates sourced for composite material production might also be agricultural side streams carrying a burden of inactive fungal material. Our protocol also allows for processing a high number of samples in parallel and enables the robust quantification of fungal biomass inside fungal composite materials.

### Substrate milling for reference cultivation

**Timing: 1 day**

1. Perform screen milling of about 10 g of each substrate that will be used for the fungal composite material production or use another suitable homogenization technique to reduce the particle size of all substrates to 1–2 mm.
2. Sieve the material and use the fine fraction (<1–2 mm) for preparation of the reference cultivation medium.
3. Dry all substrates at 60°C to constant weight. You will need about 5 g per dry substrate.

### Preparing fungal reference samples

**Timing: 5 h**

4. If using multiple substrates, calculate the ratio that will be used in the fungal composite material.
5. With these ratios, weigh the corresponding mass to reach a final solid content of 2% weight per volume into a 250 ml glass bottle that can be autoclaved (i.e., 5 g for 250 mL).
6. Add 4 g Agar-Agar (final concentration of 1.6% w/v), fill up to 250 mL and autoclave the media for 20 min at 121°C.
7. Pour about 5 petri dishes with 20 mL medium per plate and let the plates solidify.

***Note:*** Make sure to mix the medium properly before each plate as the substrate particles might separate fast.

8. Place a PES filter membrane on the medium surface. This filter will aid in separating the fungal biomass from the solidified medium after the cultivation.
9. Inoculate the fungal organism of choice on the center of the filter. Prepare 5 plates per fungi and substrate mixture to be tested.
10. Store the plates in a container with a small beaker of water (or an incubator with humidity control) to increase air humidity and to prevent plates from drying out during longer incubation periods. Otherwise, do incubate under the same conditions and for the same duration as the fungal composite material (See next step).

### Preparing fungal composite materials

**Timing: 4 weeks**

11. Prepare fungal composite materials as applicable. This usually comprises of an initial cultivation step after mixing the inoculum with the degermed/sterilized main substrate, a homogenization step followed by filling moulds and a secondary cultivation stage.

### Prepare the ergosterol standard

12. Weigh 3.96 mg Ergosterol into a 15 mL falcon tube or similar and dissolve in 10 mL of HPLC-grade methanol. This stock solution has a concentration of 1mM. Vortex vigorously until fully dissolved, this may take up to 5 min. A prior incubation of the tightly capped tube at 60°C helps to facilitate dissolving. Store at 15–25°C in the dark for up to 14 days. Wrap lid with Parafilm to prevent evaporation of MeOH.
13. On the day of HPLC analyses, prepare a dilution series using the 1 mM Ergosterol standard solution. Use pure HPLC-grade methanol for the dilutions. Reaction tubes made of polypropylene are suitable.
14. Recommended concentrations for the standard curve are 0 μM, 0.1 μM, 0.5 μM, 1 μM, 10 μM, 50 μM, 100 μM, 200 μM, 400 μM, and 600 μM.

***Note:*** The limit of detection (LOD) for Ergosterol is around 0.1 μM for most HPLC systems at an injection volume of 100 μL. This can be affected by the cell volume of the detector module.

15. Transfer 300 μL of each standard to a suitable HPLC screw-cap vial.

**CRITICAL:** Methanol is toxic (GHS06), a health hazard (GHS08) and flammable (GHS02). Use a fume hood for handling to prevent inhalation and wear PPE and nitrile gloves to prevent skin contact.

### Prepare the HPLC instrument

16. Connect a bottle of HPLC-grade methanol to the solvent inlet of the HPLC pump.
17. Mount the C18 column (see key resources table).
18. Set the temperate column of the column oven to 25°C.
19. Prepare an isocratic HPLC run program with a total time of 20 min and a flowrate of 0.8 mL/min. Set the injection volume to 100 μL per vial.
20. Set the UV-Vis detector to scan mode, when possible. This can help to verify the identity of the ergosterol. A wavelength of 280 nm corresponding to the absorbance maximum of ergosterol will be used later for quantification.

***Note:*** The expected retention time of ergosterol at a column temperature of 25°C, a flow rate of 0.8 mL/min and a 250 mm column with 4.6 mm inner diameter is approximately 14.5 min. The method can be shortened by increasing the column temperature to 30°C after ensuring there are no further peaks close to the ergosterol peak that could interfere with analysis.

## Key resources table

**Table:** 

REAGENT or RESOURCE	SOURCE	IDENTIFIER
**Chemicals, peptides, and recombinant proteins**		

Ergosterol, 98% purity	ThermoFisher Scientific	#117810050
Chloroform, HPLC grade	VWR Chemicals	#83627.320
Methanol, HPLC grade	VWR Chemicals	#152506X
Agar-Agar	N/A	N/A

**Experimental models: Organisms/strains**		

*Fomes fomentarius* PaPF11 **or own** fungal strains of interest	N/A	N/A

**Other**		

Petri dishes, 90 mm diameter with vents	N/A	N/A
Polyethersulfone (PES) filter 0.2 μm meshsize, 47 mm diamter	Merck Millipore	#GPWP04700
Parafilm	N/A	N/A
Ruler, measure tape and camera for documentation	N/A	N/A
Lyophillizer or freezer-dryer **alternative**: oven heated to 60°C	i.e., from Christ or Büchi	N/A
Band-saw for cutting samples **alternative**: fine hand saw	N/A	N/A
Precision Lab Scales with 0.1 mg readability	N/A	N/A
Fume hood for handling of solvents and extraction	N/A	N/A
Gaduated glass cylinder for measuring of solvents	N/A	N/A
Glass bottle for mixing of extraction solvent	N/A	N/A
2 ml reaction tubes with snap caps	N/A	N/A
Multi-dispenser pipette or positive displacement pipette with suitable pipette tips	i.e., from Eppendorf	N/A
Thermal mixer with shaking function for 2 mL tubes	N/A	N/A
Oven heated to 60°C	N/A	N/A
Rotational vacuum voncentrator suitable for solvent removal **alternative**: manifold for nitrogen blowdown of solvents	i.e., from Eppendorf	N/A
HPLC-System with integrated UV-VIS detector	i.e., from Shimadzu	N/A
C18-HPLC column, 250 mm length × 4.6 mm inner diameter, particle size 5 μm	Macherey-Nagel	NUCLEOSIL 100–5 C 18
Suitable glass vials and screw-caps with silicone insert for the chosen HPLC-system	i.e., from VWR	548-0386A and 548-3256A

## Materials and equipment

**Table:** Ergosterol Extraction Solution

Reagent	Final concentration	Amount
Chloroform	66%	100 mL
Methanol	33%	50 mL
**Total**	**N/A**	**150 mL**

Store at 15–25°C under a fume hood for up to 3 months.

**CRITICAL:** Chloroform and methanol are toxic (GHS06) and a health hazard (GHS08). Additionally, methanol is flammable (GHS02). Use a fume hood for handling to prevent inhalation, wear appropriate gloves to prevent skin contact and safety glasses .

## Step-by-step method details

### Sample preparation for ergosterol extraction

**Timing: 2–3 days**

This step prepares the fungal composite material and reference samples by drying to constant weight and prepares and documents the sample fractions where ergosterol is to be extracted from.

1. Harvest the fungal biomass from the reference plate by scraping the mycelium off the filter using a spatula or peel off with tweezers, when suitable.
2. Transfer the biomass to a 14 mL falcon tube and oven-dry at 60°C or preferably freeze-dry the biomass.
3. Grind the biomass to a fine powder using a spatula or cut to fine pieces (2–3 mm) using a scalpel.
4. After reaching the intended cultivation time of the fungal composite material, stop growth of fungus with appropriate method. Usually, this is accomplished by drying at temperatures around 60°C until a constant moisture content is reached.

***Note:*** For all subsequent steps, we assume that biomaterial samples are grown as a sheet with a height of 5 to 10 cm and are prepared from aggregated particles of a plant-based feedstock with a particle size between 1 and 7 mm and a fungal inoculum.

5. Use a handsaw or preferably a band saw to cut the sample into slices of 3 to 4 cm thickness. Clearly mark the orientation of the upper side of the sample to preserve the orientation.

***Note:*** If increased dust formation during sawing is expected to occur, wear a particle mask (FFP2) to prevent inhalation of the material and make sure there is adequate ventilation. Wear safety glasses while cutting and wear protection gloves.

6. On the slices, horizontal layers can be marked with tape or a pen. Photograph the sample with a ruler to document the orientation and appearance. If multiple layers are to be extracted from the sample, layers should have a height of about 25–30 mm.

***Note:*** The growth of the fungus inside a material is impacted by several factors, amongst them oxygen and nutrient availability. To monitor gradients of fungal biomass inside the material, consider using narrower slices (<10 mm) to increase the resolution.

7. Using tweezers, separate the particles of the material into workable volumes and transfer to a storage container (Falcon tube or small beaker).
8. Oven-dry at 60°C or preferably freeze-dry the samples till reaching a constant mass for accurate quantification in the subsequent steps.
9. For each slice, measure between 50–90 mg of dried composite material sample into a 2 mL reaction tube (polypropylene). Prepare at least five technical replicates per layer to be analyzed and record the mass of each sample.

***Note:*** The samples do not need to be of equal weight but within the range of 50 to 90 mg of dried composite material.

10. For pure fungal biomass from the reference plate, weight 10–25 mg dried sample into a 2 mL reaction tube (polypropylene). Prepare at least five technical replicates per sample.
11. Weigh 50 to 75 mg of dried, raw substrate material into a 2 ml reaction tube (polypropylene). Prepare at least five technical replicates per substrate.

***Note:*** It is important to include control samples of the non-colonized substrate used for biomaterial preparation. Especially when working with field-derived raw materials, previous growth of molds can lead to presence of ergosterol in detectable quantities which have to be subtracted from the samples.

### Ergosterol extraction

**Timing: 1 day**

This step extracts the ergosterol from the prepared sample material and produces samples that can be analyzed by HPLC to quantify the ergosterol in the subsequent step.

12. Work under a fume hood and add 1.5 mL ergosterol extraction solution to each tube prepared in the previous method step. Plastic pipette tips made of polypropylene (PP) are suitable.

***Note:*** Preferably, work with a positive displacement pipette or a multi-dispenser pipette. Otherwise, aspirate the ergosterol extraction solution a few times to saturate the gas phase inside the pipette before the first transfer to avoid spilling of the solution.

13. Incubate the samples for 4 h at 800 rpm and 25°C using a bench-top thermomixer placed under a fume hood.
14. Centrifuge sample for 10 min at 12.000 × *g*^-1^ to sediment finer particles.
15. Carefully aspirate 300 μL of supernatant and transfer to a new 1.5 mL reaction tube.
16. Completely evaporate the extraction solvent using a vacuum concentrator for 45 min and 45°C using the HV setting for highly volatile solvents.

***Note:*** Alternatively, the solvent can be removed under a gentle stream of nitrogen if a manifold for this is available or left to dry for at least 2 h under a fume hood in a thermomixer set to 30°C.

17. Pipette 300 μL of HPLC-grade methanol to each tube.

***Note:*** Preferably, work with a positive displacement pipette or a multi-dispenser pipette.

18. Incubate the samples for 20 min at 45°C and 800 rpm in a thermomixer placed under a fume hood to dissolve all compounds that are soluble in methanol.
19. Centrifuge the samples for 10 min at 12.000 × *g*^-1^.
20. Transfer 250 μL of each sample to a HPLC vial and proceed to ergosterol quantification.

***Note:*** Store samples tightly capped at 15–25°C temperature for not longer than 24 h to circumvent evaporation effects of methanol.

### Sample analysis via HPLC

**Timing: 20 min per sample**

This step quantifies the ergosterol in the samples using HPLC. A standard curve and reference samples are then used to calculate the abundance of fungal biomass in the composite material of interest.

21. Perform injections of 100 μL per ergosterol standard first, beginning from the lowest to the highest concentration. Include a blank injection of pure methanol after the highest standard concentration to ensure no carry-over of sample material.

***Note:*** The protocol uses an isocratic gradient and relies on the solubility of compounds in methanol after extraction, thereby limiting the potential build-up of unwanted compounds on the column over time. However, if severe performance issues are observed after 50–100 runs, consider cleaning the column with solvents (acetonitrile, isopropanol) recommended by the manufacturer for removal of contaminants such as lipids and other hydrophobic compounds. Route the flow path around the detector unit during these steps. Wear proper PPE all the time.

22. Inject the sample of pure fungal biomass and the biomaterial samples in sequential manner, including blank injections of 100 μL methanol when appropriate.

***Note:*** Compare the absorbance spectra of ergosterol peaks from the standard curve to evaluate ergosterol peak purity in the composite material samples.

### Ergosterol quantification

23. Extract the peak area of ergosterol (at an RT of 14.5 min with the HPLC conditions and column given in the KRT) for all samples from the respective HPLC chromatograms. Use the appropriate function in the HPLC software for this step. Import the peak area, retention time and sample identifier for each injection into a spreadsheet software of choice for further calculations.

**Figure 1:**
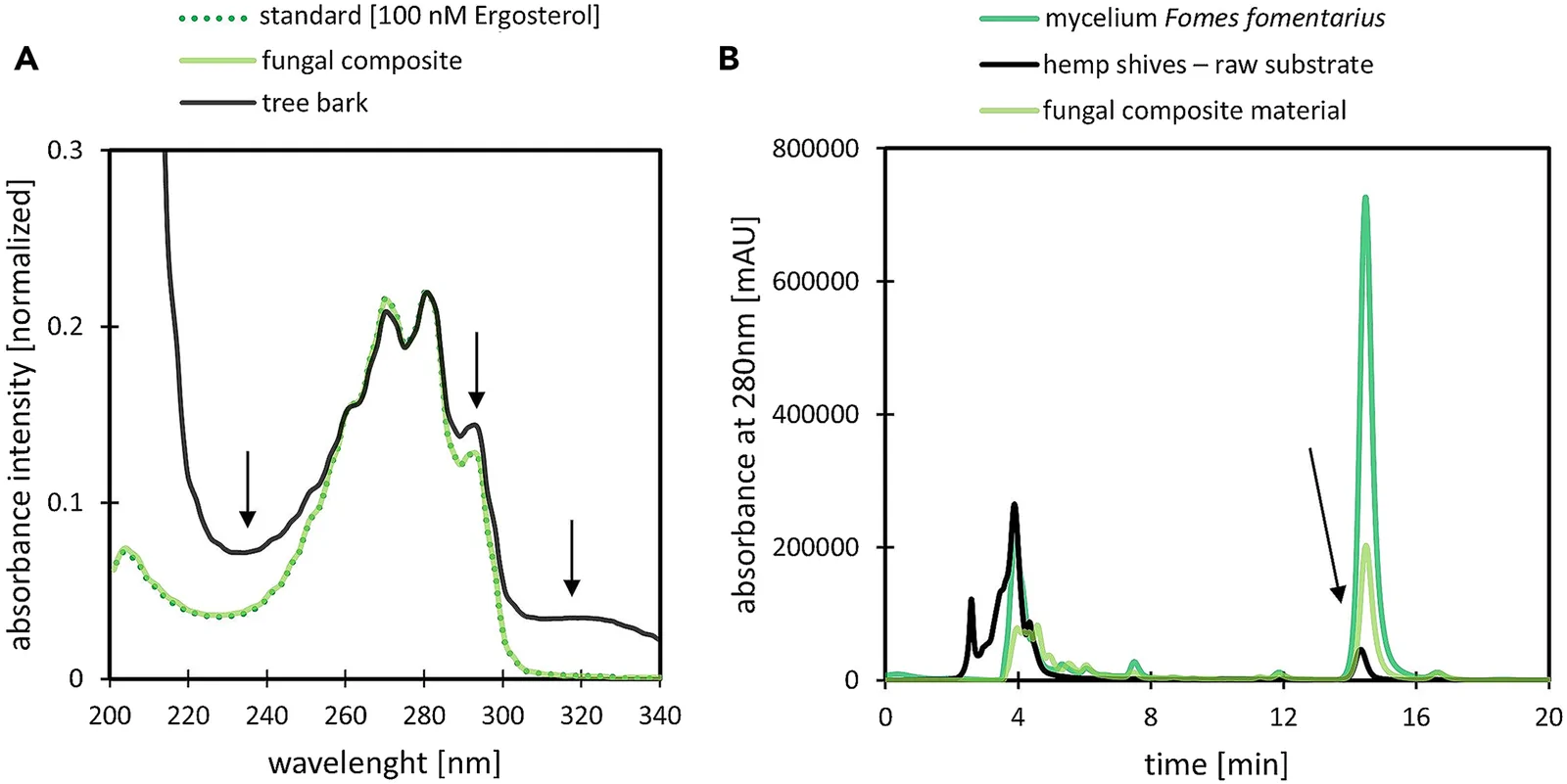
Reference spectrum of ergosterol standard and HPLC chromatogram of selected extracts of samples (A) Absorbance spectrum of a peak eluting at a retention time of 14.5 min. The spectra of the ergosterol standard (dark green dotted line) displays a maximum at 280 nm and a characteristic peak shape between 200 and 340 nm. Extract samples from fungal composite materials made from hemp shives do show an identical absorbance (light green). In contrast, extracts from tree bark do show an altered absorbance spectrum, suggesting co-elution of compounds (differences highlighted by arrows). (B) Chromatogram at an absorbance of 280 nm over the runtime of the HPLC method. Samples of pure mycelium of *Fomes fomentarius* (dark green line), a fungal composite material prepared from *Fomes fomentarius*, millet grains and hemp shives (light green line) and the raw hemp shive substrate (black line) are plotted for comparison. The peak corresponding to the elution of ergosterol from the HPLC column is highlighted with an arrow.

**Figure 2:**
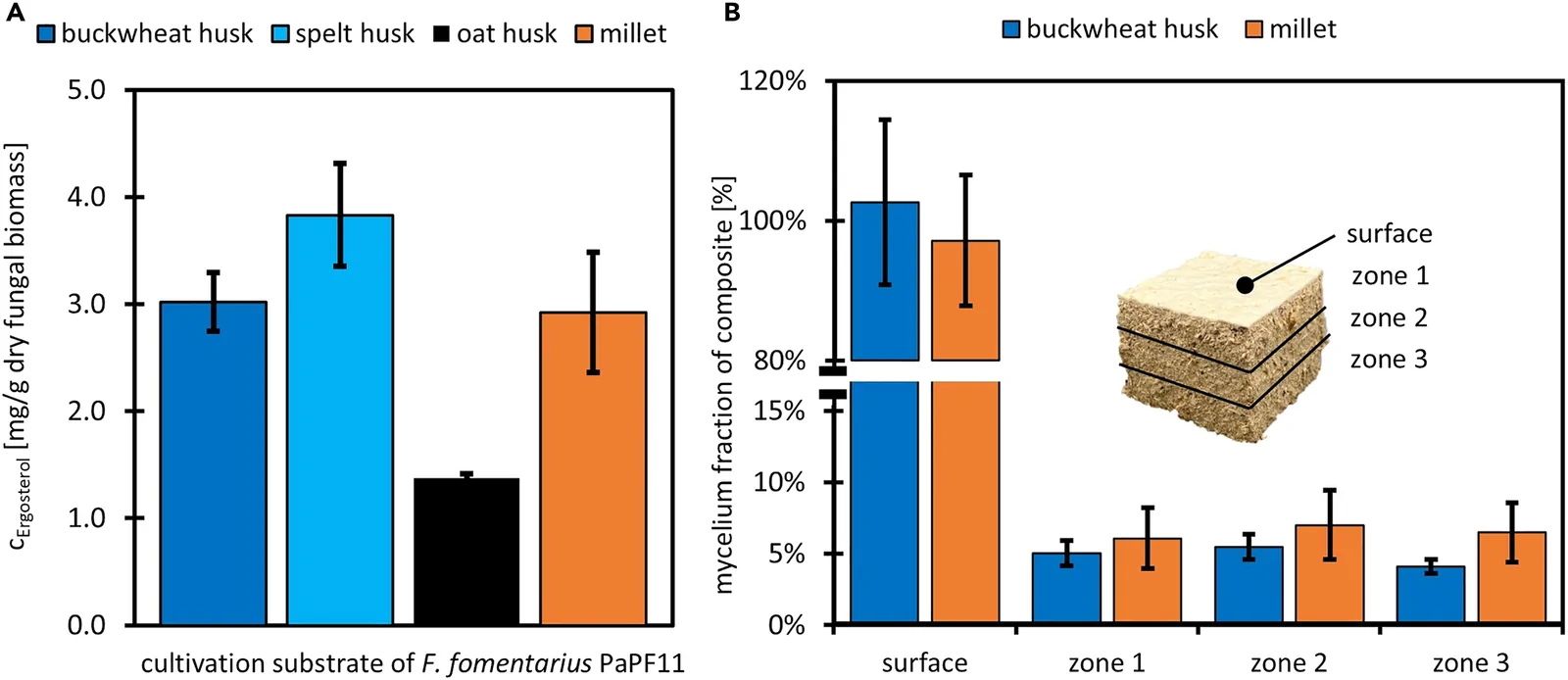
Impact of cultivation substrates on ergosterol concentration and expected outcome of this protocol (A) Ergosterol concentration of *Fomes fomentarius* PaPF11 cultivated on solid medium containing different lignocellulosic feedstocks (dark blue: buckwheat husks; light blue: spelt husks; black: oat husks; orange: brown millet) as sole carbon and nitrogen source. Error bars represent the standard deviation obtained from 5 independent extractions. (B) Distribution of fungal mycelium on the surface and in different zones of fungal composite material. Samples were produced from hemp shives inoculated with different precultures (dark blue: buckwheat husks; orange: millet). Zone height is 2.3 cm. Each error bar represents the standard deviation calculated by aggregating the data from 4 independent samples extracted in triplicate (*n* =12).

***Note:*** Compare the spectrum of the ergosterol peak from samples to the spectrum of the ergosterol standard to ensure that the purity of the peak is not compromised by co-eluting compounds. This can occur when working with samples containing a high amount of aromatic and extractable compounds, i.e. tree bark (see Figure 2A).

24. Calculate the calibration curve of ergosterol by plotting the peak areas of the used standards over their concentrations.

***Note:*** Negative control samples of the raw feedstock used for fungal composite production may also show an ergosterol signal. For instance, hemp plants are usually retted by microbial communities including fungi to facilitate separation of the fiber from the stalk, leading to detectable levels of ergosterol (see Figure 2B - hemp shives, raw substrate). If this occurs, calculate the average ergosterol concentration in the feedstock (mgErgosterol/gFeedstock) and subtract that number from the ergosterol concentration in the composite material sample ) (mgErgosterol/gComposite) to correct for previous occurrence of mold contaminations to derive the corrected concentration.

25. By using the calibration curve, do calculate the ergosterol concentration in the pure fungal samples, state this as mass of Ergosterol per mg cell dry weight (CDW) – the unit of this number is [mgErgosterol/gCDW].

***Note:*** The concentration is fungus-specific and growth-condition-specific and ranges between 0.5 and 10 mg ergosterol per g CDW.

26. By using the calibration curve, do calculate the ergosterol concentration in the biomaterial samples, state this as the mass of ergosterol per g dry composite material [mgErgosterol/gComposite].
27. Divide the ergosterol concentration of the biomaterial by the ergosterol concentration in the pure fungal biomass to obtain the fraction (in percent) of fungal biomass in the biomaterial based on the ergosterol concentration.

## Expected outcomes

This method quantifies the fraction of fungal biomass inside a mixture of organic and/or inorganic particles, which are frequently called fungal composite materials. Figure 1 presents data that were obtained applying our protocol. Depending on the lignocellulosic substrate the fungus is grown on, the ergosterol accumulation in the cell membrane is altered (Figure 1A). Extracting ergosterol from pure fungal biomass grown under the conditions and using the same media composition as in composite material production, this reference culture is very important to correctly estimate the fungal biomass (Figure 1B). The biomass fraction of the composite material extrapolated from the ergosterol concentration is preferentially represented as a bar plot and standard deviations and provides a profile of fungal biomass abundance inside a material sample.

***Note:*** Average numbers derived from at least 5 technical replicates as the variation of this method is relatively high (10–15%), which is due to different particle surface areas that are colonized by the fungus. This leads to variation, especially when a low mass of sample is analyzed and extracted If the variation has to be decreased, larger quantities of composite need to be extracted.

## Limitations

This method should not be used if a peak appears in the control that has an RT similar to that of ergosterol. Further, if plastic particles are embedded in the produced composite biomaterial or plastic coatings have been used, they might become dissolved in the extraction buffer and form aggregates when evaporating the buffer. This might negatively impact dissolving ergosterol in the subsequent step. We highly advise checking the compatibility of any plastics embedded in the material with Chloroform.

## Troubleshooting

### Problem 1

The particle size of the material used in the fungal composite is not suitable for sampling into 2 ml reaction tubes (Step 5).

### Potential solution

If the fungal composite material is dominated by larger particles (>10 mm), scale up the extraction process to larger glass vials or glass bottles and perform extraction on a rolling incubator instead of a shaker. Alternatively, the composite samples can also be milled after drying.

### Problem 2

The ergosterol peak absorbance spectra in extract samples deviates from the ergosterol standard. (Step 24).

### Potential solution

We are aware that an isocratic HPLC method cannot separate molecules that co-elute with ergosterol. We therefore suggest monitoring the spectrum of the ergosterol peak. If the spectra appear to be contaminated by other substances, reduce the flow rate to 0.5 ml/min and increase the run length to 30 min to separate potential overlapping peaks. If this does not improve separation, the isocratic protocol can be altered to a gradient with acetonitrile (1%/min from 0 to 20% acetonitrile followed by 10 min at 0% acetonitrile to reconstitute the column at a flowrate of 0.5 ml/min) to improve separation.

## Resource availability

### Lead contact

Further information and requests for resources and reagents should be directed to and will be fulfilled by the lead contact, Ulla Simon (ulla.simon@ceramics.tu-berlin.de).

### Technical contact

Technical questions on executing this protocol should be directed to and will be answered by the technical contact, Carsten Freidank-Pohl (carsten.pohl@tu-berlin.de).

### Materials availability

This protocol does not include unique materials.

### Data and code availability

This protocol does not generate datasets and includes no code.

## Acknowledgments

Open Access funding was enabled and organized by Project DEAL. This work was supported by grant 520637539 to C.F.-P. and by grant 548244816 (Collaborative Research Centre 1743, Project MY-CO BUILD) to U.S. and C.F.-P. by the Deutsche Forschungsgemeinschaft DFG. Stefan Platzk (Chair of Mechanical Process Engineering and Solids Processing [MVTA], Technische Universität Berlin) is acknowledged for screen milling of substrate samples. The graphical abstract was created using Biorender.com.

## Author contributions

C.F.-P. conceived and supervised the project. A.G. and C.F.-P. performed the experiments and wrote the experimental parts of this protocol and the troubleshooting section. C.F.-P. and U.S. co-wrote the introduction, prepared the figures and reviewed and edited the article. The final text was approved by all co-authors.

## Declaration of interests

The authors declare no competing interests.
